# Antibiofilm Agents for the Treatment and Prevention of Bacterial Vaginosis: A Systematic Narrative Review

**DOI:** 10.1093/infdis/jiae134

**Published:** 2024-04-29

**Authors:** Michael Gao, Jim Manos, Greg Whiteley, Iryna Zablotska-Manos

**Affiliations:** Faculty of Medicine and Health, Sydney Medical School, University of Sydney, Westmead, NSW, Australia; Faculty of Medicine and Health, Westmead Clinical School, Westmead, NSW, Australia; Infection Immunity and Inflammation, Faculty of Medicine and Health, School of Medical Sciences, University of Sydney, Westmead, NSW, Australia; Sydney Institute of Infectious Diseases, The University of Sydney, Westmead, NSW Australia; Infection Immunity and Inflammation, Faculty of Medicine and Health, School of Medical Sciences, University of Sydney, Westmead, NSW, Australia; Sydney Institute of Infectious Diseases, The University of Sydney, Westmead, NSW Australia; School of Medicine, Western Sydney University, Campbelltown, NSW, Australia; Whiteley Corporation, North Sydney, NSW, Australia; Sydney Institute of Infectious Diseases, The University of Sydney, Westmead, NSW Australia; Faculty of Medicine and Health, Westmead Clinical School, Westmead, NSW, Australia; Western Sydney Sexual Health Centre, Parramatta, NSW, Australia

**Keywords:** bacterial vaginosis, biofilm, prevention, recurrence, treatment

## Abstract

**Background:**

Bacterial vaginosis (BV) is difficult to eradicate due to BV biofilms protecting BV bacteria (Gardnerella, Prevotella, and other genera). With the growing understanding of biofilms, we systematically reviewed the current knowledge on the efficacy of anti-BV biofilm agents.

**Methods:**

We searched literature in the Scopus, Medline, and Embase databases for empirical studies investigating substances for the treatment of BV biofilms or prevention of their recurrence and their efficacy and/or safety.

**Results:**

Of 201 unique titles, 35 satisfied the inclusion criteria. Most studies (89%) reported on preclinical laboratory research on the efficacy of experimental antibiofilm agents (80%) rather than their safety. Over 50% were published within the past 5 years. Agents were classified into 7 groups: antibiotics, antiseptics, cationic peptides, enzymes, plant extracts, probiotics, and surfactants/surfactant components. Enzymes and probiotics were most commonly investigated. Earlier reports of antibiotics having anti-BV biofilm activity have not been confirmed. Some compounds from other classes demonstrated promising anti-BV biofilm efficacy in early studies.

**Conclusions:**

Further research is anticipated on successful antibiofilm agents. If confirmed as effective and safe in human clinical trials, they may offer a breakthrough in BV treatment. With rising antibiotic resistance, antibiofilm agents will significantly improve the current standard of care for BV management.

## Teaser

The treatment of bacterial vaginosis (BV) is complicated by the antibiotics’ inability to penetrate BV biofilms. This review informs clinicians about a variety of investigated BV biofilm removal treatments and their efficacy, as well as particularly promising recent treatment approaches with enzymes and probiotics.

Bacterial vaginosis (BV) is a neglected and poorly treated condition with a significant burden for women worldwide [1, 2]. In Australia, a BV prevalence of 12% was reported, as compared with 30% in North America and >50% in southern and eastern Africa [3–5]. Most affected are women of reproductive age [3, 6].

BV is a polymicrobial condition with a complex pathophysiology [1, 7–9] where the native vaginal microbiome, mainly *Lactobacillus* sp, is disrupted by increased numbers of Gardnerella species, such as *G vaginalis* and *G swidsinskii/leopoldi*, which grow together in biofilms with *Prevotella* species such as *P bivia* [2]. Significant adverse reproductive and obstetric outcomes are associated with BV, such as miscarriage, preterm labor, preterm rupture of membranes, chorioamnionitis, pelvic inflammatory disease, and an increased risk of acquiring sexually transmitted infections including HIV [2, 4, 10, 11].

BV treatment has changed very little since metronidazole was first introduced 40 years ago [12]. Due to teratogenic concerns with metronidazole during that time, clindamycin was added as an alternative in the late 1980s [13]. Current standard-of-care treatment with one of these antibiotics [10, 11, 14, 15] can achieve an initial cure rate between 80% and 90% [3, 7]. However, more than half of all patients experienced recurrence within 6 months and up to 80% within 9 months of treatment [1, 14]. With the general increase in antimicrobial resistance, repeated use of antibiotics for the treatment of recurrent BV is clearly unsustainable [9, 10].

Several factors are believed to play a role in the high recurrence rates, such as ineffective penetration of these antibiotics into BV biofilms and the possibility of reinfection from sexual activities [11, 14]. The recognition of bacterial biofilms in enabling the survival and growth of different species is relatively recent [16]. Evidence suggests the presence of in situ vaginal biofilms in women with BV, before and after antibiotic therapy [17, 18]. Many microbial pathogens are recognized as significant biofilm producers, and once they are inside a biofilm, resistance to all forms of stress—including antiseptics, disinfectants, and antibiotics—is increased by orders of magnitude [19, 20]. Importantly, biofilms are not just the structures adhering to surfaces: bacteria within biofilms exhibit increased cellular activity, well beyond outputs observed in docile or planktonic phases [21]. Inside a biofilm, bacteria can also achieve a dormant state, and survival is well beyond the normal expectation [22].

In recent years, the increased understanding of BV biofilms has stimulated research on how to improve the standard of care through the development of antibiofilm agents [1, 6]. Currently, a range of antibiofilm agents is being actively explored, including various antiseptics, cationic peptides, enzymes, plant extracts, probiotics, and surfactants that could assist in penetration of the biofilm structure [6, 9, 14]. However, there are no literature reviews summarizing the existing evidence from anti-BV biofilm research.

## AIM

The aim of this review is to summarize current evidence on a range of therapeutic agents with potential activity against BV biofilms.

## METHODS

This review follows the reporting guidelines of PRISMA 2020 (Preferred Reporting Items for Systematic Reviews and Meta-analyses).

### Literature Search Strategy

A systematic search for primary peer-reviewed publications was conducted in Scopus, Medline and Embase on 4 June 2023. The primary concepts were BV, biofilms, and treatment and prevention. Search terms for each concept (keywords in Scopus; Medical Subject Headings terms and keywords in Medline and Embase) were combined into the following search formula for Scopus with Boolean terms (AND and OR) and wild cards:

(ALL (treat* OR prevent* OR therap* OR disrupt* OR eradicat* OR surfactant* OR antioxidant* OR anti-biofilm* OR astodrimer* OR dendrimer* OR (boric AND acid*) OR dnase* OR lysozyme* OR antiseptic OR retrocycline* OR tol-463 OR spl7013 OR octenidine OR subtilosin OR lauramide)) AND ((KEY (biofilm*)) AND (KEY (“bacterial vaginosis” OR “vaginal dysbiosis” OR “vaginitis”))) AND (LIMIT-TO (LANGUAGE, “English”) OR LIMIT-TO (LANGUAGE, “Russian”) OR LIMIT-TO (LANGUAGE, “German”)) AND (LIMIT-TO (DOCTYPE, “ar”)) AND (LIMIT-TO (SRCTYPE, “j”)).

Equivalent search formulas were used in Medline and Embase. Citation chaining was performed on identified publications to maximize search results.

Given the relative novelty of this research area, the publication date was not limited.

### Selection of Publications

Predefined inclusion/exclusion criteria (Table 1) guided screening of identified articles. Included articles were peer-reviewed primary research publications of any study design that examined the efficacy and safety of therapeutic compounds in the treatment or prevention of BV biofilms. We included only publications in Chinese, English, German, Russian, and Ukrainian, which we could review without requiring translation.

**Table 1.** Inclusion and Exclusion Criteria for Selection of Published Articles

Inclusion Criteria	Exclusion Criteria
Treatment or prevention of bacterial vaginosis biofilms	Nonprimary research sources: review articles, editorials, opinion papers
Primary research study	Conference abstracts
Peer-reviewed publication	Ongoing clinical trials where publication is protocol only (ie, no results available)
Language of publication: Chinese, English, German, Russian, Ukrainian	Case studies, case series
Laboratory research, studies in animals or humans	Studies relating to cost-effectiveness, acceptability of treatment, attitudes to treatment
Studies of efficacy and safety on currently available antibiofilm treatments	…

Excluded were articles of nonprimary research, conference abstracts, ongoing clinical trials with no available results, and case studies or series, as well as studies of treatment cost, acceptability, or attitudes. Also excluded were articles that did not report extractable data pertaining to the aim of this review.

**Figure 1.**
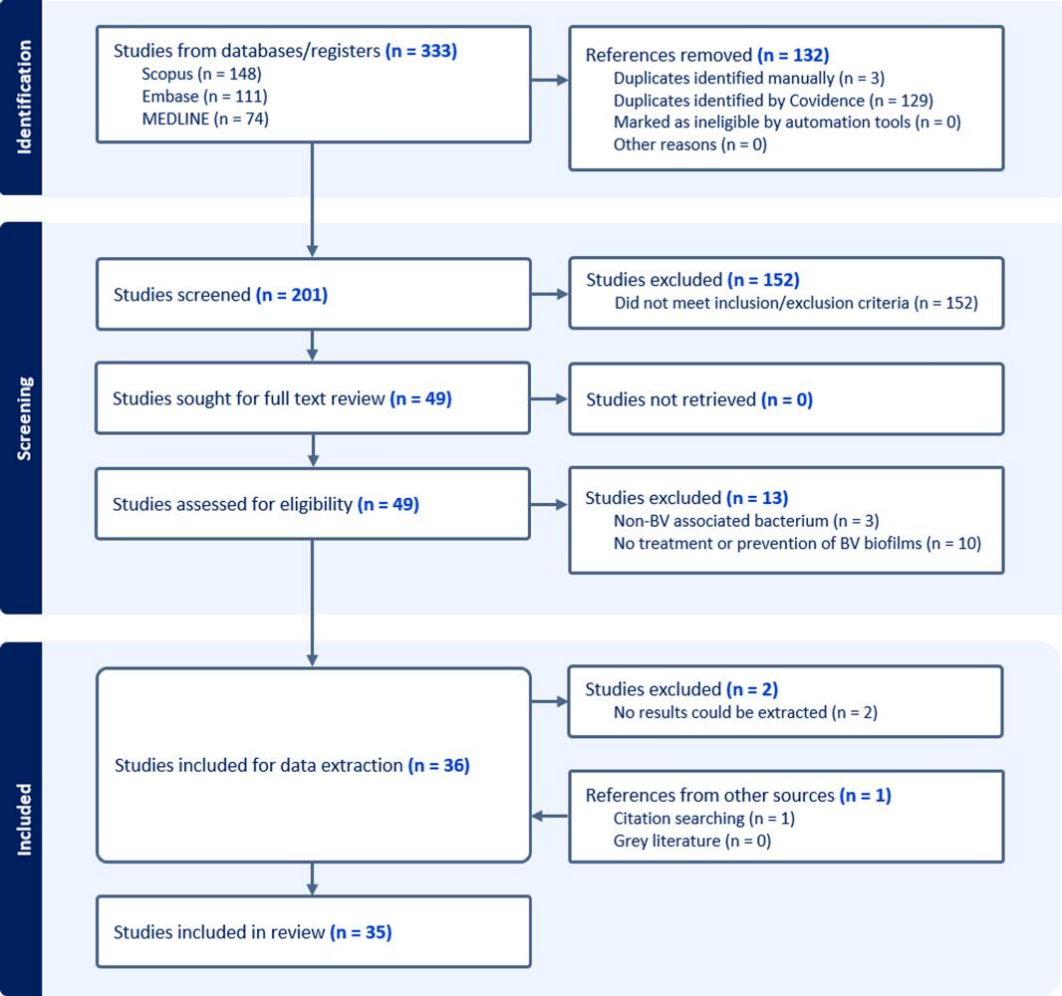
PRISMA flowchart of literature search. Adapted from Covidence. BV, bacterial vaginosis; PRISMA, Preferred Reporting Items for Systematic Reviews and Meta-Analyses.

Primary screening of titles, abstracts, and full text was performed by 2 reviewers using Covidence systematic review software (Veritas Health Innovation; Covidence.org). Disagreements were discussed and finalized. See PRISMA flowchart (Figure 1) for our selection process.

### Data Extraction

Data were extracted into Microsoft Excel. Four groups of variables were extracted: publication description (journal, year of publication, country), study aim (prevention or eradication, population, age range, biofilm species), research method (study design, sample size, therapeutic agent, comparator, mechanism of action, single agent or combination therapy, efficacy measurement, safety measurement), and research results.

Quality appraisal of the articles was conducted with the ARRIVE 2.0 guideline for animal research, the Cochrane Risk of Bias 2 tool for a randomized clinical trial, the Newcastle-Ottawa quality assessment tool for cohort studies, and the modified MICRO framework for cell culture studies. Results are available on request.

## RESULTS

### Literature Search Results

Of 333 articles identified (Figure 1), 201 unique titles and abstracts were screened against inclusion/exclusion criteria (Table 1), and 49 proceeded to full-text review. Of those, 13 were excluded due to the investigation of non–BV-associated bacteria or for not studying BV-specific biofilms. Two articles [23, 24], despite meeting all selection criteria, were additionally excluded during data extraction as their results could not be adequately extracted. Citation chaining yielded 1 additional eligible reference [25], bringing the final number of publications to 35.

### Characteristics of Included Publications

All articles were published in English, starting from 1998. Over 50% (n = 18) were published in the past 5 years (January 2017–May 2023). Of 35 publications, 18 originated in Europe (Germany and Portugal, 5 each; United Kingdom, 3; Austria and Italy, 2 each; Belgium, 1), 9 from North America (United States, 7; Canada, 2), and 8 from Asia (China, 6; Korea and Taiwan, 1 each).

The studies were predominantly carried out on laboratory cell cultures, reflecting the early stage of research (Table 2). The types of research facilities conducting experiments were not described; however, the author affiliations suggest a variety of settings, such as university, hospital, and industrial research laboratories. Experimental agents were predominantly used alone and mostly to eradicate BV biofilms rather than prevent their formation. Nine studies [26, 34–41] sought to evaluate the efficacy of the investigative therapeutic in combination with other antimicrobials (predominantly standard-of-care antibiotics metronidazole or clindamycin). Only 4 studies in humans were identified, all on small cohorts of adult premenopausal women (sample size range, 18–44). See Supplementary Appendix 1 for additional details of study characteristics.

**Table 2.** Study Characteristics (N = 35)

Study Characteristic	No.	%
Study design		
Laboratory cell culture	31	89
Animal (mice)^a^	5	14
Human^b^	4	11
Human studies only, range		
Sample size	18–44	…
Age, y	20–51	…
Eradication and prevention of biofilm		
Eradication only	19	54
Prevention only	5	14
Eradication and prevention	11	31
Treatment approach		
Therapeutic agent only	26	74
Therapeutic agent combined with other antimicrobials	9	26

^a^These 5 publications [26–30] are also counted under “laboratory cell culture,” as the authors worked with animals (mice) and cell cultures.

^b^Contains 1 randomized clinical trial [31] comparing treatment and control groups. The other studies [18, 32, 33] are single-arm interventional studies where all participants received treatment.

Therapeutic agents were classified into 7 groups based on their mechanisms of action (Supplementary Appendix 2). Antibiotics were investigated in approximately a quarter of the publications (n = 9) [17, 18, 32, 34, 37, 42–45]. Enzymes (n = 8) [26, 36, 37, 40, 46–49], cationic peptides (n = 7) [27, 34, 35, 37, 41, 45, 50], probiotics (n = 7) [25, 28, 29, 38, 39, 51, 52], antiseptics (n = 5) [30, 33, 37, 53, 54], and surfactants or their components (n = 2) [31, 37] were also frequently investigated as potential anti-BV biofilm treatments. Most studies (n = 28, 80%) investigated treatment efficacy, while the remaining 20% (n = 7) additionally evaluated safety. Given the lack of standardization in measuring efficacy outcomes, these were broadly grouped into 5 categories: biofilm cell viability (n = 20), biofilm biomass (n = 15), direct growth inhibition (n = 14), biofilm morphology (n = 6), and molecular quantification (n = 3). Safety was measured in only 7 studies, and measures of safety were grouped into 3 mutually exclusive categories: vaginal epithelial cell changes (n = 4) [27, 30, 40, 41], tolerance of lactobacilli to therapeutics (n = 2) [35, 55], and adverse events and tolerability (human studies, n = 1) [31].

Findings, including specific agents within each therapeutic class, are summarized in Table 3. Seven preclinical studies investigated the effect of antibiotics on preformed in vitro biofilms: amoxicillin [43], clindamycin [17, 42–44], erythromycin [43], metronidazole [17, 37, 42–44], subtilisin A [34, 45], and tobramycin [37]. Earlier studies reported significant efficacy of 2 antibiotics against preformed single-species *G vaginalis* biofilms, including complete biofilm eradication with clindamycin [43] and a 2-log reduction [45] or complete biofilm eradication [34] with subtilisin. However, no such effect with clindamycin was observed in more recent in vitro studies on single-species *G vaginalis* biofilms [17, 42] and a triple-species biofilm (*G vaginalis*, *Fannyhessea vaginae*, and *Peptostreptococcus anaerobius*) [44] . Two additional human cohort studies measuring the effect of metronidazole [18] and moxifloxacin [32] on in vivo BV biofilms also noted a lack of efficacy.

**Table 3.** Treatment Efficacy and Safety by Treatment Class and Aim

		No. of Publications by Treatment Aim^a^					
Treatment		Prevention		Eradication		Safety	
Class	Agent	Yes	No	Yes	No	Yes	No
Antibiotics							
A1	Amoxycillin				1 [43]		
A2	Clindamycin		1 [42]	1 [43]	3 [17, 42, 44]		
A3	Erythromycin				1 [43]		
A4	Metronidazole	1 [37]	1 [42]		6 [17, 18, 37, 42–44]		
A5	Moxifloxacin				1 [32]		
A6	Subtilosin A			2 [34, 45]			
A7	Tobramycin	1 [37]			1 [37]		
Antiseptics							
B1	Benzoyl peroxide	1 [53]					
B2	Cetylpyridinium chloride	1 [37]		1 [37]			
B3	Chlorocresol	1 [37]		1 [37]			
B4	Dequalinium chloride	1 [54]					
B5	Iron sulfide D-Fe_3_S_4_	1 [30]				1 [30]	
B6	Octenidine				1 [33]		
B7	Polyaminopropyl biguanide		1 [37]		1 [37]		
Cationic peptides							
C1	Cationic amphiphiles 1a, 1b, 1c, 2a, 2b, 2c	1 [41]		1 [41]		1 [41]	
C2	Cationic amphiphiles G8, G10	1 [35]		1 [35]		1 [35]	
C3	ε-Poly-L-lysine (polylysine)			1 [45]			
C4	Lauramide arginine ethyl ester			2 [34, 45]			
C5	Linear cationic peptide OP145		1 [37]		1 [37]		
C6	Retrocyclin RC-101	1 [50]			1 [50]		
C7	Tilapia piscidin 4 peptide	1 [27]		1 [27]		1 [27]	
Enzymes							
D1	DNase	1 [26]		1 [26]			
D2	Endolysins CCB^b^		1 [36]	2 [36, 47]			
D3	Endolysin PM-477			3 [46, 48, 49]			
D4	Lysozyme	1 [37]		2 [37, 40]		1 [40]	
D5	Proteinase K	1 [37]		1 [37]			
Plant extracts							
E1	Essential oil (from *Thymbra capitata*)			2 [55, 56]		1 [55]	
E2	Salicylic acid	1 [53]					
E3	Thymol (from *Thymus vulgaris*)	1 [57]		1 [57]			
Probiotics							
F1	*Lacticaseibacillus casei*	1 [51]					
F2	*Lactobacillus crispatus*			1 [52]			
F3	*Lactobacillus gasseri*	1 [29]					
F4	*Lactobacillus helveticus*	1 [28]					
F5	*Lactobacillus iners*				1 [52]		
F6	*Lactobacillus plantarum*	1 [25]		1 [25]			
F7	*Lactobacillus reuteri*			2 [38, 52]			
F8	*Lacticaseibacillus rhamnosus*	2 [39, 51]		2 [38, 39]	1 [52]		
F9	*Saccharomyces cerevisiae*	1 [39]		1 [39]			
Surfactants/surfactant components							
G1	Amphoteric tenside WO3191			1 [31]		1 [31]	
G2	Lecithin		1 [37]		1 [37]		
G3	Sodium cocoamphoacetate	1 [37]		1 [37]			
Combination treatments							
	A2 + A6			1 [34]			
	A2 + C1			1 [41]			
	A2 + C4			1 [34]			
	A2 + D4			1 [40]			
	A2 + F9				1 [39]		
	A4 + A6			1 [34]			
	A4 + C1			1 [41]			
	A4 + C2	1 [35]		1 [35]			
	A4 + C4			1 [34]			
	A4 + D1			1 [26]			
	A4 + D2			1 [36]			
	A4 + D4			1 [40]			
	A4 + F9			1 [39]			
	A4 + G4			1 [37]			
	A7 + G4				1 [37]		
	F7 + F8			1 [38]			
	G2 + G4			1 [37]			

^a^ *Yes* and *no* refer to whether the result is statistically significant.

^b^CCB2M94_8, CCB2.2, CCB4.1, CCB7.1, CCB8.1.

Four preclinical studies investigated the efficacy of antiseptics: benzoyl peroxide [53], cetylpyridinium chloride [37], chlorocresol [37], dequalinium chloride [54], iron sulfide (D-Fe_3_S_4_) [30], and polyaminopropyl biguanide [37]. A significant reduction in biofilm formation was noted by all compounds except polyaminopropyl biguanide. The largest effect in this class was seen with iron sulfide, where a 4-log reduction in colony-forming units across biofilms was reported after 3 hours of exposure [30]. One human cohort study measured the effect of octenidine [33] on established in vivo biofilms. Despite initial efficacy of octenidine in biofilm disruption, 37% of the study participants developed resistance with repeated exposure to the antiseptic.

Seven preclinical studies investigated cationic peptide agents: cationic amphiphiles 1a, 1b, 1c, 2a, 2b, and 2c [41] as well as G8 and G10 [35], polylysine [45], lauramide arginine ethyl ester [34, 45], cationic peptide OP145 [37], retrocyclin RC-101 [50], and tilapia piscidin 4 peptide [27]. Reduction in biofilm formation was noted for cationic amphiphiles as well as for retrocyclin RC-101 and tilapia piscidin 4 peptide. Disruption of established biofilms was observed for cationic amphiphiles, polylysine, lauramide arginine ethyl ester, and tilapia piscidin 4 peptide. However, an earlier investigation of OP145 revealed no significant effect on formation or disruption of biofilms [37].

Eight preclinical studies evaluated enzymes: DNase [26], endolysins CCB (CCB2M94_8, CCB2.2, CCB4.1, CCB7.1, CCB8.1) [36, 47], endolysin PM-477 [46, 48, 49], lysozyme [37, 40], and proteinase K [37]. Significant reduction of preformed biofilms was achieved by all compounds tested in this class. In particular, endolysin PM-477 was reported to have eliminated all in vitro biofilms after 24 hours of exposure [48]. The same research group also noted a lesser but still significant 75% reduction on ex vivo BV biofilms [49]. Dnase, lysozyme, and proteinase K prevented biofilm formation but not endolysins CCB.

Four preclinical studies evaluated plant extracts: essential oil [55, 56], salicylic acid [53], and thymol [57]. All compounds were effective in disrupting preformed biofilms. Notably, in a study of a 6-species BV biofilm containing *G vaginalis*, *F vaginae*, *Lactobacillus iners*, *Mobiluncus curtisii*, *P anaerobius*, and *P bivia*, exposure to essential oil for 24 hours resulted in eradication of all biofilm-associated bacteria in vitro and a 4-log reduction ex vivo [56]. An early study also showed effectiveness of thymol in *G vaginalis* biofilm prevention [57].

Seven preclinical studies investigated 9 probiotic species: *Lacticaseibacillus casei* [51], *Lactobacillus crispatus* [52], *Lactobacillus gasseri* [29], *Lactobacillus helveticus* [28], *Lactobacillus iners* [38], *Lactobacillus plantarum* [25], *Lactobacillus reuteri* [38, 52], *Lacticaseibacillus rhamnosus* [38, 39, 51], and *Saccharomyces cerevisiae* [39]. Inhibition of biofilm formation was reported in studies with *L casei*, *L gasseri*, *L helveticus*, *L plantarum*, and *S cerevisiae*. Significant disruption of existing biofilms was observed with *L crispatus*, *L plantarum*, *L reuteri*, and *S cerevisiae* but not *L iners*. Two recent articles [38, 39] also noted significant efficacy of *L rhamnosus* in disrupting preformed biofilms, in contrast to an earlier study [52] that found no effect.

In a preclinical study, Gottschick et al evaluated the surfactant sodium cocoamphoacetate and the surfactant component lecithin [37]. Efficacy in prevention and disruption of biofilms was reported for sodium cocoamphoacetate but not lecithin. In a randomized clinical trial by the same research group, efficacy in disrupting in vivo biofilms was indicated for an amphoteric tenside (WO3191) [31].

Nine preclinical studies tested 17 combination therapies against preformed biofilms. The majority (n = 14) of these combinations tested the investigative agent with clindamycin [34, 39–41] or metronidazole [26, 34–37, 39–41]. All combinations tested showed greater efficacy against preformed biofilms as compared with individual use of the investigative agent, except for the combination of *S cerevisiae* and clindamycin where no greater efficacy was observed [39]. Two other effective combinations were *L reuteri* with *L rhamnosus* [38] and cetylpyridinium chloride with sodium cocoamphoacetate [37]. Reduced efficacy was noted when sodium cocoamphoacetate was combined with tobramycin [37].

Although there were no safety concerns described for any of the therapeutic agents, it is worth mentioning that treatment safety was assessed in only 7 of the 35 articles [27, 30, 31, 35, 40, 41, 55].

## DISCUSSION

Various potential BV biofilm removal treatments are currently being actively investigated. We grouped these treatments into 7 classes: antibiotics, antiseptics, cationic peptides, enzymes, plant extracts, probiotics, and surfactants/surfactant components. Within the last 5 years, enzymes and probiotics were most investigated, although these substances are still in early stages of development with their efficacy yet to be demonstrated through human trials. The growing knowledge about BV biofilms is anticipated to stimulate research in this area.

In vivo biofilms contain multiple BV-associated bacterial species [7, 9, 14, 58, 59], but some are nonculturable in the laboratory setting. In general, laboratory studies tended to use single-species *G vaginalis* biofilms, which does not reflect the multispecies biofilm conditions in vivo. This limitation was noted by a number of authors within their articles [25, 26, 28, 30, 39, 42, 44, 55]. Several publications attempted to use multispecies biofilm models [36, 38, 40, 44, 46, 47, 56], noting a higher treatment resistance when compared with single-species biofilms. In addition, safety and tolerability of the treatment were not adequately measured in most studies. Future research should not only prioritize the testing of these agents in biofilm models that better approximate in vivo conditions but also measure safety and tolerability through clinical trials.

Antibiotic treatments have always been the standard of care for BV [10, 11, 14, 15]. Narrow-spectrum antibiotics have a selective mechanism of action and target specific species of bacteria [60] while minimally affecting other bacterial populations. Although several earlier publications suggested some evidence of antibiotic efficacy in combination with compounds such as subtilosin and lauramide arginine ethyl ester against preexisting biofilms [34, 43, 45], recent research found no significant anti-BV biofilm effect when they are used in isolation; therefore, the anti-BV biofilm activity of antibiotics could not be confirmed. This finding is consistent with the high failure rates and frequent recurrence of BV observed in clinical practice after treatment with metronidazole and clindamycin [5, 6, 10].

In contrast to antibiotics, antiseptics work by destroying bacteria nonselectively via multiple targets, and they affect the biofilm structure itself [60]. While these compounds were generally reported effective against BV biofilm formation in vitro, a cohort study by Swidsinskii et al [33] detected BV biofilm resistance against the antiseptic octenidine with repeated application of the treatment, which significantly reduced its efficacy over a short period. The development of resistance is a major concern not only for antiseptics but across all classes of therapeutics, and further research should aim to thoroughly assess this risk so that only the agents demonstrating low resistance potential are selected for clinical trials.

From the probiotics class, numerous *Lactobacillus* species showed promise in prevention of biofilm formation by other species and disruption of their established biofilms. An early study [52] found no significant effect from *L iners* and *L rhamnosus*; however, subsequent studies using *L rhamnosus* reported biofilm prevention and disruption potential against pathogenic BV species [38, 39, 51]. Another study [39] demonstrated efficacy against *G vaginalis* biofilms by *S cerevisiae*, especially when combined with standard-of-care antibiotics. Notably, the duration of BV biofilm exposure to the probiotic treatment was relatively short, in most instances ≤24 hours, before the experiments were stopped for efficacy measurements. Whether and for how long the observed effects can be sustained remains unknown. The duration of exposure is an important consideration when selecting an agent from any treatment class for further development, as those with sustained activity will offer a treatment advantage in the clinical setting but may potentiate side effects that warrant further testing. In this regard, the lack of a standard animal BV model has complicated efforts to identify the safety concerns of these new therapeutic agents.

Enzymes targeting bacterial cell walls, proteins, and extracellular DNA have attracted increasing attention over the past several years. Landlinger et al [48, 49], using phage endolysin PM-477, demonstrated significant efficacy against in vitro and ex vivo biofilms. These molecules were noted to have low resistance formation and are highly specific against *G vaginalis*, representing a potential safety benefit that should be examined in future studies.

Various compounds from other classes demonstrated significant preclinical anti-BV biofilm efficacy. Earlier studies with cationic peptides OP145 [37] and RC-101 [50] showed no effect in disrupting existing biofilms; however, a number of newer agents in this class demonstrated efficacy in prevention and disruption of BV biofilms through reduced colony-forming unit counts and biofilm viability testing. In comparison, very few studies were conducted with plant extracts. While the preliminary results on biofilm disruption with essential oil [55, 56] were promising, further clinical studies are needed to confirm its efficacy and safety.

The use of surfactants against BV biofilms has been investigated by only 1 study: a human randomized clinical trial of the amphoteric tenside WO3191 [31]. This study did report efficacy of the compound against in vivo biofilms, and the paucity of clinical research in this class of antibiofilm agents prevents any conclusive recommendation.

Our selection process excluded several other human trials that investigated the substances from the same classes described here, but they measured only the return or otherwise of symptoms posttreatment (first inclusion criterion). Based on the assumption that their efficacy was due to the disruption of BV biofilms, it is worth mentioning these trials here. A retrospective chart review conducted by Reichman et al [61] of 58 women with recurrent BV revealed that boric acid, an antiseptic, when combined with antibiotic treatment showed improved patient outcomes. More recently, in a single-blinded randomized clinical trial, Marrazzo et al [62] demonstrated the efficacy and safety of boric acid as a single-agent treatment for BV, with modest benefits of boric acid alone in the absence of antibiotics. Similarly, Surapaneni et al [63] reported a satisfactory response in a clinical noncomparative study of 105 women when boric acid was combined with antibiotic therapy, although the effect was only a mild improvement over antibiotics alone. While the mechanism of action of boric acid against BV biofilms is not fully understood, it has been suggested that this agent may inhibit their formation by influencing bacterial metabolite processes or host immune responses [64]. Further research on the effects of boric acid is needed, especially on its anti-BV biofilm properties.

Noteworthy, the ongoing search for novel antibiofilm agents against BV echoes the progress made in the treatment of biofilms in other anatomic locations. For instance, respiratory biofilm research in cystic fibrosis shares many treatment similarities and classes of compounds, such as antibacterials/antibiotics, biofilm matrix–disrupting agents, quorum-sensing inhibitors and intracellular signaling suppressors, and iron metabolism disruptors [65–68]. Additionally, there have been reports of a range of probiotics and plant extracts being successfully used against oral biofilms [69]. While many differences exist among these types of biofilms, including anatomy, bacterial species, and overall diversity, it is possible that advances in biofilm treatment in these areas may contribute to the research and development of new anti-BV biofilm treatments.

### Strengths and Limitations of This Review

To our knowledge, this is the first review to detail current research evidence on different anti-BV biofilm treatments. These findings inform clinicians about developments in the field and in turn offer their patients hope of a long-term cure.

Several limitations of this review should be acknowledged. While publication bias was not specifically assessed, it was noted that the majority of publications reported on effective compounds. Quite likely, significant research work that has found investigative compounds ineffective remains unpublished and was consequently not captured by this review. Additionally, the heterogeneity of study designs made comparisons among findings difficult since observations in laboratory cultures and animal models are not often reflected in human subject research.

## CONCLUSION

The ongoing search for new therapeutics against BV has been complicated by an incomplete understanding of its pathophysiology and the role of biofilms. Numerous promising agents are being investigated, and their findings indicate that the application of anti-BV biofilm agents may very likely bring a potential breakthrough in the treatment of BV; however, this is still an evolving field predominantly in the preclinical stages of research. With concerns of antibiotic resistance, action is urgently needed to move the study of these diverse antibiofilm agents into animal and human trials, although the absence of optimal animal models for BV is slowing the momentum of this much-needed development.

## Supplementary Data

Supplementary materials are available at *The Journal of Infectious Diseases* online (http://jid.oxfordjournals.org/). Supplementary materials consist of data provided by the author that are published to benefit the reader. The posted materials are not copyedited. The contents of all supplementary data are the sole responsibility of the authors. Questions or messages regarding errors should be addressed to the author.

## Supplementary Material

jiae134_Supplementary_Data

## Notes

***Authors contributions.*** M. G. conducted database searches, screened publications, extracted data, and prepared the draft manuscript. J. M. reviewed the draft manuscript and provided guidance on the classification of antibiofilm agents and their mechanisms of action. G. W. reviewed the draft manuscript and provided guidance on the classification of antibiofilm agents and their mechanisms of action. I. Z.-M. supervised the project, screened publications, and reviewed and edited the draft manuscript.

***Financial support*.** No financial support was received for this work.
