# Supplementary material for: Antibiofilm Agents for the Treatment and Prevention of Bacterial Vaginosis: A Systematic Narrative Review
Source: J Infect Dis. 2024 Apr 29;230(3):e508–17. doi: 10.1093/infdis/jiae134 (PMC11420799; doi:10.1093/infdis/jiae134)
Supplement: jiae134_Supplementary_Data [file jiae134_supplementary_data.docx]

**APPENDICES**

**Appendix 1: List of included publications and their characteristics.**

| **REF #^[[1]](#footnote-1)^** | **Author, year of publication** | **Country** | **Population^[[2]](#footnote-2)^** | **Sample Size (Human Trials)** | **Prevention (P) or Eradication (E)** | **Safety (S) or Efficacy (EF)** | **Stage Preclinical (PC) or Clinical (C)** | **Study Design^[[3]](#footnote-3)^** | **Biofilm studied** | **Therapeutic Agent** | **Combination Therapy (Therapeutic Agent in combination with)** |
| --- | --- | --- | --- | --- | --- | --- | --- | --- | --- | --- | --- |
| 31 | Algburi et al., 2017 | USA | CC |  | E | EF | PC | LLC | In-vitro | Lauramide arginine ethyl ester; Subtilosin | Clindamycin; Metronidazole |
| 59 | Algburi et al., 2018 | USA | CC |  | P,E | EF, S | PC | LLC | In-vitro | Cationic amphiphiles G8 and G10 | Metronidazole |
| 30 | Algburi, Volski, Chikindas, 2015 | USA | CC |  | P | EF | PC | LLC | In-vitro | Benzoyl peroxide; Salicylic acid | No |
| 32 | Arroyo-Moreno et al., 2022 | UK | CC |  | P,E | EF | PC | LLC | In-vitro | Endolysins CCB2M94_8, CCB7.1, CCB8.1, CCB2.2, and CCB4.1 | Metronidazole |
| 63 | Braga et al., 2010 | Italy | CC |  | P,E | EF | PC | LLC | In-vitro | Thymol | No |
| 46 | Castro et al., 2022 | Portugal | CC |  | E | EF | PC | LLC | In-vitro | Endolysin PM-477 | No |
| 56 | Fang et al., 2022 | China | CC, A (mice) |  | E | EF, S | PC | LLC, aTC | In-vitro, in-vivo | Iron sulfide D-Fe_3_S_4_ | No |
| 60 | Gaspar et al., 2021 | Portugal | CC |  | E | EF | PC | LLC | In-vitro | Dequalinium chloride | No |
| 33 | Gottschick et al., 2016 | Germany | CC |  | P,E | EF | PC | LLC | In-vitro | Cetylpyridinium chloride; Chlorocresol; Lecithin; Lysozyme; Linear cationic peptide OP145; Metronidazole; Polyaminopropyl biguanide; Proteinase k; Sodium cocoamphoacetate; Tobramycin | Sodium cocoamphoacetate: Cetylpyridinium chloride; Metronidazole; Tobramycin |
| 58 | Gottschick et al., 2017 | Germany | F (ages 19-51) | 44 | E | EF, S | C | hRCT | In-vivo | Amphoteric tenside WO3191 | No |
| 52 | He et al., 2021 | China | CC |  | P | EF | PC | LLC | In-vitro | *L. casei; L. rhamnosus* | No |
| 50 | Hooven et al., 2012 | USA | CC |  | P,E | EF | PC | LLC | In-vitro | Retrocyclin RC-101 | No |
| 34 | Hymes et al., 2013 | USA | CC, A (mice) |  | P,E | EF | PC | LLC, aTC | In-vitro,  in-vivo | DNase | Metronidazole |
| 47 | Johnston et al., 2023 | UK | CC |  | E | EF | PC | LLC | In-vitro | Endolysin CCB7.1 | No |
| 53 | Kim et al., 2023 | South Korea | CC,A (mice) |  | P | EF | PC | LLC, aTC | In-vitro,  in-vivo | *L. helveticus* | No |
| 49 | Landlinger et al., 2021 | Austria | CC |  | E | EF | PC | LLC | In-vitro,  ex-vivo (ages 24-49) | Endolysin PM-477 | No |
| 48 | Landlinger et al., 2022 | Austria | CC |  | E | EF | PC | LLC | In-vitro | Endolysin PM-477 | No |
| 39 | Li et al., 2020 | China | CC |  | E | EF | PC | LLC | In-vitro | Clindamycin; Metronidazole | No |
| 51 | Lin et al., 2022 | Taiwan | CC, A (mice) |  | P,E | EF, S | PC | LLC, aTC | In-vitro,  in-vivo | Tilapia piscidin 4 peptide | No |
| 40 | Ma et al., 2022 | China | CC |  | P,E | EF | PC | LLC | In-vitro | Clindamycin; Metronidazole | No |
| 57 | Machado et al., 2017 | Portugal | CC |  | E | EF, S | PC | LLC | In-vitro | *T. capitata* essential oil | No |
| 35 | McMillan et al., 2011 | Canada | CC |  | E | EF | PC | LLC | In-vitro | *L. reuteri; L. rhamnosus* | Metronidazole |
| 41 | Muli and Struthers, 1998 | UK | CC |  | E | EF | PC | LLC | In-vitro | Amoxicillin; Clindamycin; Erythromycin; Metronidazole | No |
| 29 | Qian et al., 2021 | China | CC |  | P,E | EF | PC | LLC | In-vitro | *L. plantarum* | No |
| 62 | Rosca et al., 2022a | Portugal | CC |  | E | EF | PC | LLC | In-vitro, ex-vivo^[[4]](#footnote-4)^ | *T. capitata* essential oil | No |
| 42 | Rosca et al., 2022b | Portugal | CC |  | E | EF | PC | LLC | In-vitro | Clindamycin; Metronidazole | No |
| 36 | Sabbatini et al., 2020 | Italy | CC |  | P,E | EF | PC | LLC | In-vitro | *L. rhamnosus; S. cerevisiae* | Clindamycin; Metronidazole |
| 54 | Saunders et al., 2007 | Canada | CC |  | E | EF | PC | LLC | In-vitro | *L. crispatus; L. iners; L. reuteri; L. rhamnosus* | No |
| 44 | Swidsinski et al., 2008 | Germany | F (mean age=26.3) | 18 | E | EF | C | hLNC | In-vivo | Metronidazole | No |
| 43 | Swidsinski et al., 2011 | Germany | F (ages 20-47) | 20 | E | EF | C | hLNC | In-vivo | Moxifloxacin | No |
| 61 | Swidsinski et al., 2015 | Germany | F (ages 22-44) | 24 | E | EF | C | hLNC | In-vivo | Octenidine | No |
| 37 | Thellin et al, 2016 | Belgium | CC |  | E | EF, S | PC | LLC | In-vitro | Lysozyme | Clindamycin; Metronidazole |
| 45 | Turovskiy et al., 2012 | USA | CC |  | E | EF | PC | LLC | In-vitro | Lauramide arginine ethyl ester; Polylysine; Subtilosin | No |
| 38 | Weeks et al., 2019 | USA | CC |  | P,E | EF, S | PC | LLC | In-vitro | Cationic amphiphiles 1a, 1b, 1c, 2a, 2b and 2c | Clindamycin; Metronidazole |
| 55 | Zhang et al., 2022 | China | CC, A (mice) |  | P | EF | PC | LLC, aTC | In-vitro, in-vivo | *L. gasseri* | No |

**Appendix 2: General mechanism of action for each treatment class**

| **Treatment class** | **Mechanism of action** |
| --- | --- |
| Antibiotics | Molecules that selectively target a component within bacterial cells,^70^ depending on the type of antibiotic. Can inhibit protein synthesis, nucleic acid synthesis, or disrupt cell membrane integrity.^36, 39, 45, 47, 48^ |
| Antiseptics | Substances that non-selectively destroy bacteria via multiple targets.^70^ Reactive oxygen species generation, quorum sensing inhibition, disrupting bacterial adhesion, disrupting cell membrane, enzymatic inactivation, ferroptotic damage (by iron sulfides), preventing bacterial aggregation.^61, 63, 64^ |
| Cationic peptides | Net positive charge of molecules neutralises negatively charged bacterial membrane and biofilm surfaces, causing cell clumping, disruption to protein synthesis and increasing antibiotic susceptibility.^36, 37, 39, 50, 55, 56^ |
| Enzymes | Enzymatic degradation of bacterial cell wall, proteins, and extracellular DNA.^38-40, 52, 53^ |
| Plant extracts | Hydrophobic disruption of cell membrane, induce cytoplasmic coagulation, prevents bacterial aggregation and adhesion to vaginal epithelium.^62, 63, 67^ |
| Probiotics | Not well described. Likely to involve multiple mechanisms, including reduction of available nutrients, competitive inhibition of attachment sites on vaginal epithelial cells, quorum sensing inhibition, production of antimicrobials, enzymes, biosurfactants, bacteriocins, reduction in virulence factor expression and sialidase activity of *G. vaginalis* required for biofilm formation.^34, 41, 42, 57-60^ |
| Surfactant/surfactant components | Hydrophilic and hydrophobic domains with emulsification properties to disrupt cell membranes.^39^ |

1. Reference number in the manuscript [↑](#footnote-ref-1)
2. CC- cell culture, F - Female patients, A - animals [↑](#footnote-ref-2)
3. LLC, longitudinal laboratory experiments with controls; LLNC, aTC, animal trials with controls; hRCT, randomised clinical trial in humans; hLNC human longitudinal study with no control (intervention arm only) [↑](#footnote-ref-3)
4. The study used reconstructed human vaginal epithelium for the ex-vivo experiments. [↑](#footnote-ref-4)
